# 96-Well Agarose-Gel Electromembrane Extraction

**DOI:** 10.1021/acs.analchem.5c06783

**Published:** 2025-12-22

**Authors:** Thidarat Samkumpim, Samira Dowlatshah, Waleed Alahmad, Pakorn Varanusupakul, Helena Hruskova, Frederik André Hansen, Stig Pedersen-Bjergaard

**Affiliations:** † Department of Pharmacy, 6305 University of Oslo, P.O. Box 1068, Blindern, 0316 Oslo, Norway; ‡ Department of Chemistry, 26683 Chulalongkorn University, Patumwan, Bangkok 10330, Thailand; § Extraction Technologies Norway, Verkstedveien 29, 1424 Ski, Norway; ∥ Department of Chemistry, University of Oslo, P.O. Box 1033, 0315 Oslo, Norway; ⊥ Institute of Analytical Chemistry of the Czech Academy of Sciences, Brno 602 00, Czech Republic; # Department of Pharmacy, Faculty of Health and Medical Sciences, University of Copenhagen, Universitetsparken 2, 2100 Copenhagen, Denmark

## Abstract

For the first time, gel electromembrane extraction was demonstrated in a 96-well system. Gel membranes of 3% w/v agarose and with a thickness of 3.5 mm were immobilized in hydrophilic polyvinylidene fluoride (PVDF) filters in a 96-well filter plate. The filters provided mechanical support for the gel membranes, and were important for the stability and robustness of the system. A selection of 90 basic pharmaceuticals in the polarity range −4.2 < log *P* < 8.1 was used as model analytes (compounds). The compounds were extracted from 200 μL sample (water or human plasma) adjusted to pH 4.0 with dilute formic acid, through the gel membrane, and into 200 μL of 100 mM formic acid as acceptor. The extraction potential was 25 V, and the extraction time was 20 min for careful operation to limit Joule heating and electroendosmosis. The majority of the compounds in the polarity range −4.0 < log *P* < 3.0 were extracted with high recovery (40–100%). Compounds with log *P* > 3.0 were discriminated due to interactions with the gel membrane. Proteins and phospholipids were not extracted, and the system therefore provided efficient cleanup from human plasma samples. The 96-well agarose-gel electromembrane extraction (EME) system showed great potential. Selectivity was controlled by interactions with the aqueous gel membrane. This is fundamentally very different from traditional EME with oil membranes, where selectivity is controlled by electro-assisted partition in and out of the oil membrane. 96-Well electromembrane extraction with gel membranes of agarose is favorable in terms of greenness and efficiency for polar analytes, as compared with systems based on oil membranes.

## Graphical abstract

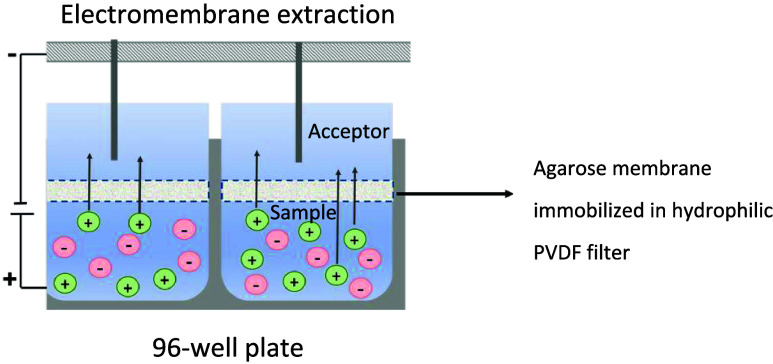

## Introduction

Electromembrane extraction (EME) is a microextraction technique and was introduced in 2006.^1^ EME involves transfer of analytes with positive or negative charge from an aqueous sample, through an organic liquid membrane, and into an aqueous acceptor forced by an electrical potential. In traditional EME, the liquid membrane is an organic solvent (membrane solvent), immobilized by capillary forces in a porous polymeric membrane. 2-Nitrophenyl octyl ether (NPOE) is commonly used as a membrane solvent for EME of basic analytes. NPOE is hydrophobic (log *P* = 4.9) and immiscible with water, and is not leaking into the aqueous sample and acceptor.^2^ NPOE is aromatic and provides hydrogen bond acceptor properties, and solvates protonated amines by hydrogen bond and cation–π interactions. NPOE extracts cationic analytes with log *P* in the range 2.0–6.0.^2^ Compounds with log *P* < 2.0 are blocked by NPOE. For EME of more polar substances, deep eutectic solvents (or other less hydrophobic liquids with strong hydrogen acceptor bond properties) are used as membrane solvent, containing an ionic carrier.^3^ For the EME of acids, the electrical field is reversed, and a liquid membrane with hydrogen bond donor properties is used. EME is highly selective and provides very clean extracts. After extraction, the aqueous acceptor can be analyzed directly using liquid chromatography-mass spectrometry (LC-MS) or related instrumental techniques. As a miniaturized and environmentally friendly sample preparation technique, EME utilizes microliter volumes of sample and organic solvent, making the technique widely applicable to biological matrices such as plasma, urine, and saliva.^4^

To improve the throughput, 96-well EME has been proposed.^5^ The 96-well EME format employs a commercial 96-well filter plate with hydrophobic polyvinylidene fluoride (PVDF) filters to support and hold the liquid membrane.^6^ This setup allows the simultaneous extraction of up to 96 samples. 96-Well EME has been studied with various basic and acidic drugs.^7,8^

The hydrophobicity of NPOE makes it poorly suitable for the extraction of polar bases, and therefore alternative liquid membranes have been developed. One such membrane (termed B3) is based on a mixture of 6-methylcoumarin, thymol, 2-undecanone, and di­(2-ethylhexyl) phosphate (DEHP) as liquid membrane, which is less hydrophobic than NPOE and offers stronger interactions with polar analytes. The extraction window of this liquid membrane ranged from −2.5 < log *P* < 4.5.^9^

Gel electromembrane extraction (gel-EME) represents another approach toward polar analytes. Originally presented in 2017, gel-EME replaced the organic liquid membrane with an agarose gel.^10,11^ Agarose, a biodegradable natural biopolymer derived from algae, consists of a hydrophilic polymeric network with flexible pore size.^12^ It has excellent gelling properties, facilitating the formation of film-based layers suitable for the diffusion and electrokinetic migration of ions. Importantly, the aqueous/hydrophilic nature of the gel makes it permeable to hydrophilic analytes, and it thus offers great potential to further expand the applicability of EME. Gel-EME is applicable to various sample types, including environmental waters, biological fluids, and pharmaceuticals,^13–17^ and has been applied to both organic and inorganic compounds^18–21^ in different low-throughput formats. However, gel-EME is affected by electroendosmosis (EEO), where water moves within the system. Also, the greater permeability of the gel membrane may enable coextraction of matrix components, but this has so far not been documented well in the literature.

In the current work, we developed for the first time a 96-well system for gel-EME. This purely aqueous system was operated with an agarose gel as membrane (agarose system), and was benchmarked against a traditional 96-well EME system using NPOE and B3 as organic liquid membranes. This enabled comparison of the two different approaches under very similar conditions. Experimental recovery data were obtained for 90 different basic pharmaceuticals (−4.2 < log *P* < 8.1), extracted both from water and plasma samples. From this comprehensive set of data, the extraction performance and the extraction window were obtained. The agarose system was also evaluated in terms of current, pH stability, and electroendosmosis, and for the cleanup of inorganic salts, proteins, and phospholipids.

## Experimental Section

### Chemicals and Reagents

The model analytes were all basic pharmaceuticals obtained from Sigma-Aldrich (St. Louis, MO). Agarose was purchased from Thermo Scientific (Germany). 2-Nitrophenyl octyl ether (NPOE), 6-methylcoumarin, thymol, 2-undecanone, di­(2-ethylhexyl phosphate) (DEHP), acetonitrile (LC-MS grade), dimethyl sulfoxide (DMSO), methanol (LC-MS grade), and formic acid (LC-MS grade) were purchased from Sigma-Aldrich. MQuant pH 0.0–6.0 indicator strips were purchased from Merck KGaA, Darmstadt, Germany. All chemicals and reagents were of analytical reagent grade, unless otherwise noted.

### Solutions and Samples

Ninety basic model analytes (with log *P* values ranging from −4.2 to 8.1) were dissolved individually in methanol, DMSO, or water (1.0–9.0 mg/mL level). All of these were mixed and diluted to obtain a common solution of all 90 compounds at 5 μg/mL was prepared (stored at −28 °C). This was used for spiking samples to 10 and 50 ng/mL for traditional gel-EME and 96-well EME, respectively.

Human, drug-free citrate plasma was obtained from Oslo University Hospital (Oslo, Norway) and stored at −28 °C. Before extraction, the plasma was diluted with 100 mM formic acid to ensure all the model analytes were present as protonated species. Ultrapure water was obtained with a Millipak (0.22 μm filter) Milli-Q water purification system (Molsheim, France). Liquid membrane solvent B3 was prepared as previously reported.^3^

### 96-Well Gel-Electromembrane Extraction (Agarose System)

The equipment for 96-well EME included two main parts described in detail previously (Figure 1):^22^ a 96-well sample plate machined in stainless steel (not commercially available) and an easy-to-use 96-well filter plate with hydrophilic PVDF membrane (Multiscreen-HV, 0.45 μm pores, Merck Millipore Ltd., Carrigtwohill, Ireland). The sample plate was machined in-house in stainless steel; the total dimensions of the plate were 123 × 81 × 11 mm^3^, and it contained 96 wells, each drilled with a diameter of 8 mm and a depth of 10 mm. The wells in the sample plate served as a reservoir for the samples. The sample plate was conductive (stainless steel) and served as a positive electrode. The agarose membrane was fabricated according to the previous work.^19^ Pure agarose powder corresponding to 3% w/v was dissolved in Milli-Q water and heated until fully dissolved. The solution was pipetted on a 96-well filter plate above the hydrophilic PVDF membrane. The solution was completely immersed in the hydrophilic PVDF membrane, achieving a thickness of approximately 3.5 mm. Then, the 96-well filter plate with agarose solution (pH approximately 6.0) was placed in a refrigerator at 4 °C until the agarose solution was solidified (approximately 2 h).

**Figure 1:**
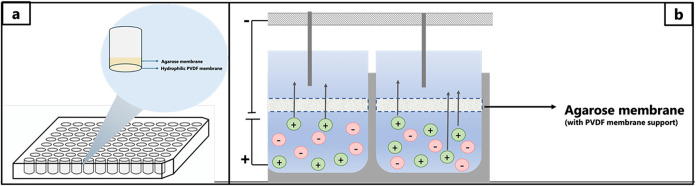
Schematics of 96-well agarose-gel electromembrane extraction.

The sample and acceptor solution volumes were 200 and 100 μL, respectively. The sample solutions were pipetted into the sample plate, and the acceptor solutions were pipetted on the 96-gel plate above the agarose gel. Then, the 96-gel and the sample plate were clamped together. The positive electrode was connected to the sample plate, and negative electrodes of platinum wires (0.5 mm, 99.9%, K.A. Rasmussen, Hamar, Norway) were inserted into the acceptor wells through a rubber stopper (one electrode per well). The 96-well EME was placed on a shaking board (Vibramax 100, Heidolph, Kellheim, Germany) operating at 900 rpm, the sample plate and electrodes were connected to a power supply (model ES 0300-0.45, Delta Elektronika BV, Zierikzee, Netherlands), and voltage was applied. Current was monitored using a Fluke 287 multimeter (Everett, WA). After the extraction, the acceptor solution was collected for ultra-high performance liquid chromatography-tandem mass spectrometry (UHPLC-MS/MS) analysis.

### Traditional Gel-Electromembrane Extraction

The agarose membrane was fabricated in the same way as described above. 400 μL of the hot agarose solution was rapidly transferred to a 1.5 mL Eppendorf tube (Hamburg, Germany) and placed in a refrigerator at 4 °C for 4 h until the solution solidified. Before the extraction, the agarose gel in the Eppendorf tube was cut to a conical shape with an optimized gel thickness.

The donor/sample solution (10 mL) was transferred to a 12 mL vial. The Eppendorf tube containing the agarose-gel membrane was placed in the vial. Then, 500 μL of the acceptor solution was pipetted above the agarose gel. The positive Pt electrode (anode) was placed in the donor vial, and the negative electrode (cathode) was placed in the acceptor solution. Both electrodes were connected to a power supply. During the extraction process, the solution was stirred at a controlled speed for a specified extraction time and the current was monitored. After complete extraction, the acceptor solution was collected for analysis with a UHPLC-MS/MS.

### Traditional 96-Well Electromembrane Extraction

For a 96-well EME using an organic solvent as a liquid membrane, the equipment and procedure were the same as described above, differing by utilizing a 96-well plate with hydrophobic PVDF filters (Multiscreen-IP, 0.45 μm pores, Merck Millipore Ltd., Carrigtwohill, Ireland). Preparation of the liquid membrane was done immediately prior to extraction by pipetting 4 μL of solvent to the bottom of the filter (facing the sample), which penetrated the pores within 30 s.

### Liquid Chromatography-Tandem Mass Spectrometry

UHPLC-MS/MS analysis was performed with an Agilent 1290 Infinity II UHPLC system (Agilent Technologies, Santa Clara, CA), consisting of a binary pump, an autosampler, and a column compartment with a controllable temperature. An Acquity UPLC HSS T3 column (50 × 2.1 mm^2^, 1.8 μm; Waters Corp., Wexford, Ireland) was used for separation. The column temperature was 40 °C, and the injection volume was 1.0 μL. Gradient elution was employed, with mobile phases A and B comprising ultrapure water, formic acid, and acetonitrile in 94.9:0.1:5 (v/v/v) and 5:0.1:94.1 (v/v) ratios, respectively, and using the following timetable: 0.00–1.00 min (0% B), 1.01–6.00 min (0–53% B), 6.01–7.00 min (75% B), 7.01–7.50 min (100% B), 7.51–9.00 min (0% B). The mobile phase flow rate was maintained at 0.4 mL/min from 0.00 to 7.00 min, increased to 0.7 mL/min between 7.01 and 8.50 min, and then returned to 0.4 mL/min from 8.51 to 9.00 min.

Mass spectrometric detection was performed with a model 6495 LC/TQ (Agilent Technologies) with positive electrospray ionization at 3.0 kV and with a desolvation gas temperature of 200 °C and 14 L/min. Nebulizer pressure was 40 psi, nozzle voltage was 1500 V, and sheath gas was delivered at 250 °C and 12 L/min. The system was operated in dynamic multiple reaction monitoring (MRM) mode with a cycle time of 300 ms, resulting in a minimum dwell time of 4.52 ms. MRM parameters are provided as Supporting Information (Table S1). The total run time was 9 min.

### Determination of Phospholipids

Acceptors were analyzed for phospholipids using the same LC-MS instrument, column, mobile phases, and injection volume as described above. The column temperature was 60 °C. Mobile phase flow rate was 0.7 mL/min, and gradient elution was applied according to the following timetable: 0.00 min (15% B), 0.00–5.00 min (0–100% B), 5.00–16.00 min (100% B), 16.10–17.00 min (15% B).

Mass spectrometric detection was performed using positive electrospray ionization at 5.5 kV and with a desolvation gas temperature of 290 °C at 12 L/min. Nebulizer pressure was 30 psi, nozzle voltage was 1800 V, and sheath gas was delivered at 400 °C and 12 L/min. The instrument operated in precursor scan mode, monitoring product ion *m*/*z* 184 corresponding to the phosphatidylcholine residue. The collision energy was set to 30 V, and precursors were scanned from *m*/*z* 200–1000 at 800 ms scan time.

### Determination of Inorganic Ions

To investigate inorganic substances in the gel-EME system, hydroxide (OH^–^) and copper­(II) (Cu^2+^) were selected as models of anionic and cationic species, respectively. In the study of OH^–^, a phenolphthalein solution was pipetted into the acceptor, and sodium hydroxide solution was pipetted into the sample. The electrodes were placed in the opposite direction. The negative electrode was applied on the sample plate, and the positive electrode was applied on the acceptor plate. Due to the transfer of OH^–^, the acceptor turned pink during extraction.

For the study of Cu^2+^, copper­(II) sulfate solution was pipetted into the sample and sodium hydroxide was pipetted into the acceptor. The positive electrode was applied in the sample, and the negative electrode was located in the acceptor. Due to the transfer of Cu^2+^, the acceptor turned light blue during extraction.

### Gel Electrophoresis (Sodium Dodecyl Sulfate-Polyacrylamide Gel Electrophoresis, SDS-PAGE)

Proteins were investigated by SDS-PAGE gel electrophoresis. With each run, a PageRuler Prestained Protein Ladder, 10–180 kDa (from Thermo Fisher Scientific) was applied. To 30 μL of the sample,10 μL of Bolt LDS sample buffer (4×) was added prior to heating at 70 °C for 10 min. The samples were loaded onto a Bolt 4–12% 2-(bis­(2-hydroxyethyl) amino)-2-(hydroxymethyl) propane-1,3-diol­(bis-tris) plus gel inserted in a mini gel tank. The chamber was filled with Bolt 2-(*N*-morpholino) ethanesulfonic acid (MES) SDS running buffer (20×), diluted to 1× with water. All Bolt products and the mini gel tank were purchased from Thermo Fisher Scientific. For 20 min, a voltage of 200 V was applied to the gel, and the gel was washed four times with water for 5 min on a shaker. Following the wash, the gel was covered with Imperial protein stain (from Thermo Fisher Scientific) and left shaking for 15 min. Before photographing the gel using a smartphone camera, the gel was washed with water four times for 5 min, followed by washing with gentle shaking in water overnight (18 h).

## Results and Discussion

The 96-well gel electromembrane extraction setup with agarose gel as the membrane (termed the agarose system) is illustrated in Figure 1. Samples were pipetted into a laboratory-built stainless steel 96-well sample plate (conductive). The sample plate was connected to the anode (positive charge) of the power supply. The corresponding agarose-gel membranes were prepared in hydrophilic PVDF filters in a commercial 96-well filter plate, and the acceptors were loaded in the reservoirs above the filters. Each acceptor reservoir was sealed with a rubber stopper, and this was perforated by a platinum wire, which was connected to the power supply and served as the cathode. During extraction, the entire setup was agitated to promote convection. The model analytes were extracted as protonated species from the samples, through the corresponding agarose-gel membranes, and into their acceptors. Since the acceptors were aqueous, they were injected directly into UHPLC-MS/MS. A mixture of 90 different pharmaceuticals was used as model analytes (compounds). All the compounds were bases, and covered the polarity range −4.2 < log *P* < 8.1 (Table S2). Their basic p*K*_a_-values ranged between 1.6 and 11.5, and to make sure the compounds were protonated and influenced by the electrical field, both the samples and acceptors were acidified with formic acid. Recovery values exceeding 40% were defined as high, and extractions were considered exhaustive when recoveries exceeded 85%.

### Optimization of the Agarose-Gel Membrane

Initial experiments with the agarose system served to optimize the extraction conditions. First, gel membranes of 2, 3, and 4% (w/v) agarose were tested. Recoveries were highest with 2% w/v agarose, but the system was more stable with 3 or 4% w/v (data not shown). As a compromise between system efficiency and stability, 3% (w/v) agarose was selected. The thickness of the agarose-gel membrane was 3.5 mm. Gel membranes thinner than 3.5 mm provided a less stable system, while those exceeding 3.5–4 mm limited the volume available for the acceptor.

### Optimization of Operational Parameters

Next, extractions were performed from samples with different concentrations of formic acid in pure water (pH range 2.0–5.0). The optimal pH was found at 4.0. As pH was reduced from 5.0, the basic compounds were fully protonated, and recoveries increased (data not shown). However, below pH 4.0, the extraction efficiency was reduced due to an unfavorable ion balance. Ion balance is defined as the ratio of the total amount of ions in the acceptor and sample, respectively.^23,24^ Therefore, the samples were adjusted to pH 4.0 with 100 mM formic acid before extraction. Acceptor pH was studied in the same range by using different dilutions of formic acid in pure water. At pH 2.0, the compounds were fully protonated, and the highest recoveries were obtained at this pH value using 100 mM formic acid as the acceptor.

Furthermore, recoveries increased with an increasing extraction potential up to 25 V. Above this level, the system was prone to serious electroendosmosis. For this reason, 25 V was used as the extraction potential. In a similar way, recoveries increased with increasing extraction time of up to 20 min. Extractions exceeding 20 min suffered from electroendosmosis (discussed below). Therefore, the extraction time was set to 20 min.

Extractions were conducted with agitation at 900 rpm (optimization data are not shown). Agitation improved the mass transfer, and the gels were mechanically stable during the extraction process because they were immobilized in hydrophilic PVDF filters.

### Extraction from Samples of Dilute Formic Acid

Recoveries with the agarose system were then measured under the optimized conditions discussed above and plotted as a function of log *P* in Figure 2a. Each data point represented a specific compound. As seen from the data, the highest recoveries were obtained for the compounds in the polarity range −2.0 < log *P* < 3.0. Within this range, 54 compounds were studied. Exhaustive extraction was achieved for 15 of these, while the remaining 39 compounds were highly scattered; 21 compounds were extracted with recoveries in the range 40–85%, while 18 compounds were below 40%. For the compounds with log *P* > 3.0, recoveries decreased rapidly with increasing log *P*, and substances with log *P* > 5.0 were not extracted.

**Figure 2:**
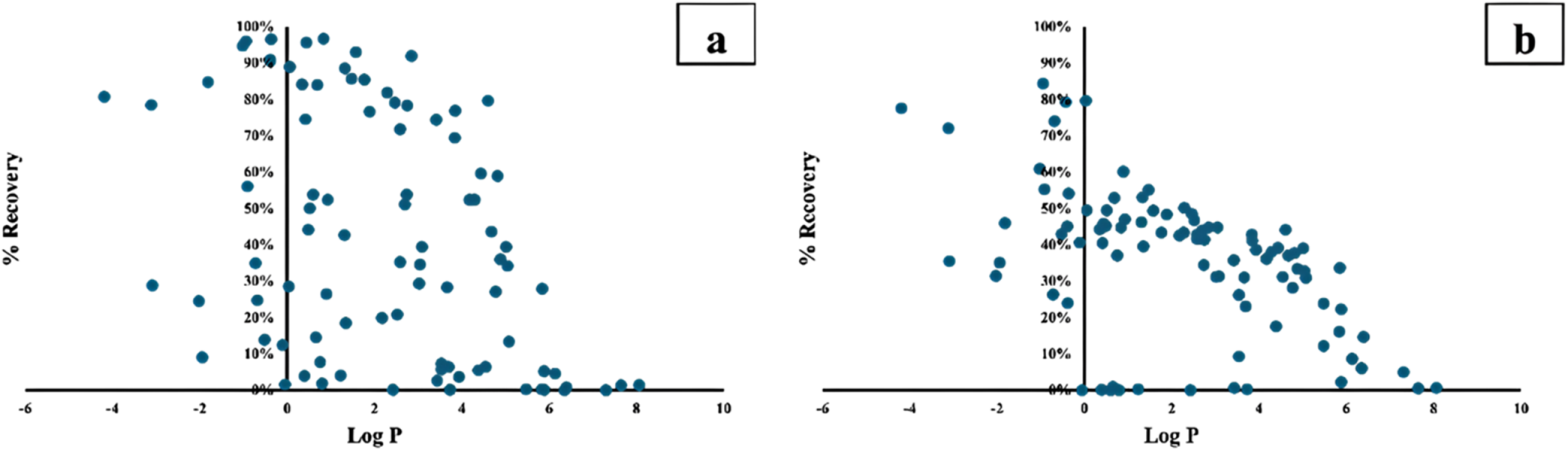
Recovery versus log *P* for 90 basic model analytes; (a) 96-well agarose-gel EME, and (b) traditional agarose-gel EME.

The recovery experiment was repeated under comparable conditions with a traditional setup for gel electromembrane extraction.^10^ The agarose-gel membrane used in the 96-well setup was immobilized in a hydrophilic PVDF filter, and the membrane thus comprised both agarose and hydrophilic PVDF. In contrast, the gel membrane in the traditional setup was pure agarose. The data in Figure 2a,b, for the 96-well setup and the traditional setup, respectively, showed similar trends, with the highest performance in the polarity range −2.0 < log *P* < 3.0. Extraction was generally more efficient with the 96-well setup. Dimensions, volumes, and diffusion distances were smaller in this setup, which explained the superior performance. The 96-well setup provided more selectivity, and the data points were more scattered. This we explained by interactions with hydrophilic PVDF, which was used for immobilization of the gel membrane only in the 96-well setup.

Compared with traditional EME using NPOE as the liquid membrane (NPOE system, Figure S1 in Supporting Information^22^), the agarose system was clearly more efficient for polar analytes. On the other hand, in the polarity range 2.0 < log *P* < 6.0, the NPOE system extracted the majority of compounds exhaustively, while the agarose system was less efficient.

The agarose system was also compared with traditional EME using a generic liquid membrane for polar bases in the polarity range −2.0 < log *P* < 4.0 (B3 system, Figure S2 in Supporting Information^3^). The B3 membrane was based on a ternary mixture of 6-methylcoumarin, thymol, 2-undecanone, and di­(2-ethylhexyl) phosphate. Within the polarity window −2.0 < log *P* < 4.0, the B3 system provided higher recoveries than the agarose system.

### Electrolysis and Electroendosmosis

During EME, electrolysis occurred at the electrodes according to the following reactions

1 $$a\mathrm{node}:2H_{2}O\to 4H^{+}+O_{2}+4e^{-}$$

2 $$c\mathrm{athode}:2H_{2}O+2e^{-}\to 2{\mathrm{OH}}^{-}+H_{2}$$

Theoretically, the pH should decrease in the sample and increase in the acceptor. The pH value was measured in the sample and acceptor before and after extraction in the agarose, B1, and B3 systems, respectively. In all cases, the changes in pH were less than 0.5 units, and electrolysis caused no practical problems.

Electroendosmosis was encountered in the agarose system. When operated at 25 V for 20 min, the transfer of water from the sample to acceptor was less than 10 μL. However, when the agarose system was operated at higher extraction potentials, or for longer time, volume changes increased significantly. With the solvent-based systems (NPOE and B3), no volume changes were measured and electroendosmosis was absent due to the organic liquid membrane.

### Extraction from Human Plasma

In the next set of experiments, extractions with the agarose system were conducted from human plasma samples spiked with the 90 compounds. Initially, the plasma samples (100 μL) were diluted 1:1 (v/v) with 100 μL of 100 mM HCOOH to ensure the compounds were protonated. However, with this sample, the current was very high upon application of 25 V, and plasma proteins precipitated in the system. Protein precipitation occurred even when the extraction potential was reduced from 25 to 5 V. In subsequent experiments, plasma diluted 1:5 and 1:10 v/v was tested. In the former case, protein precipitation was still an issue. With 10 times dilution, no protein precipitation was observed, and recoveries improved significantly (Table S2, Supporting Information). Plasma dilution may be avoided if the plasma proteins are precipitated prior to extraction.

Extraction recoveries from plasma were similar to those from pure water samples, and compounds extracted with high recovery from water, were also extracted efficiently from diluted plasma. For the same reason, the extraction window with agarose was the polarity range −4.0 < log *P* < 3.0 both from water and plasma samples. Extraction recoveries from plasma with the NPOE and B3 systems are found in Table S2 in the Supporting Information. In these experiments, the plasma volume was 100 μL, 10 times larger volume than used above with the agarose system.

### Study of Major Matrix Constituents

Human plasma samples are highly complex due to matrix constituents, such as inorganic salts, proteins, and phospholipids. In the next set of experiments, the distribution of these was studied at the end of extraction. Sodium hydroxide solution was added to the sample to study the anionic species. Phenolphthalein solution was added to the acceptor, and the reversed extraction potential (anode in the acceptor) was applied. By visual inspection, the acceptor turned pink during 5 min of operation, and this demonstrated migration of OH^–^ across the agarose-gel membrane. A similar experiment was performed with copper (Cu^2+^) ions in the sample and OH^–^ ions in the acceptor. Upon application of the electrical potential, the acceptor turned blue, and Cu^2+^ migrated across the agarose-gel membrane. Although no other inorganic ions were studied, most inorganic ions likely passed the agarose-gel membrane. The same experiments were performed in the NPOE and B3 systems, but OH^–^ and Cu^2+^ remained in the sample during extraction, as expected.

Next, proteins were studied. A plasma sample was diluted 1:10 v/v with 100 mM formic acid and processed with the agarose system. Followingly, the acceptor was analyzed for proteins by SDS-PAGE electrophoresis as illustrated in Figure 3. Lane 1 showed all of the proteins in the diluted plasma sample before extraction. SDS-PAGE of the acceptor after extraction was illustrated in lane 3. No substances above 10 kDa were detected, and the acceptor was clearly free from proteins. They were clearly discriminated in the agarose system due to strong interaction with the agarose-gel membrane, and the system provided extracts free of plasma proteins. The same experiment was performed with the NPOE and B3 systems, in Figure 3, lanes 2 and 4, respectively. These lanes were very similar to lane 3 and showed highly efficient removal of proteins.

**Figure 3:**
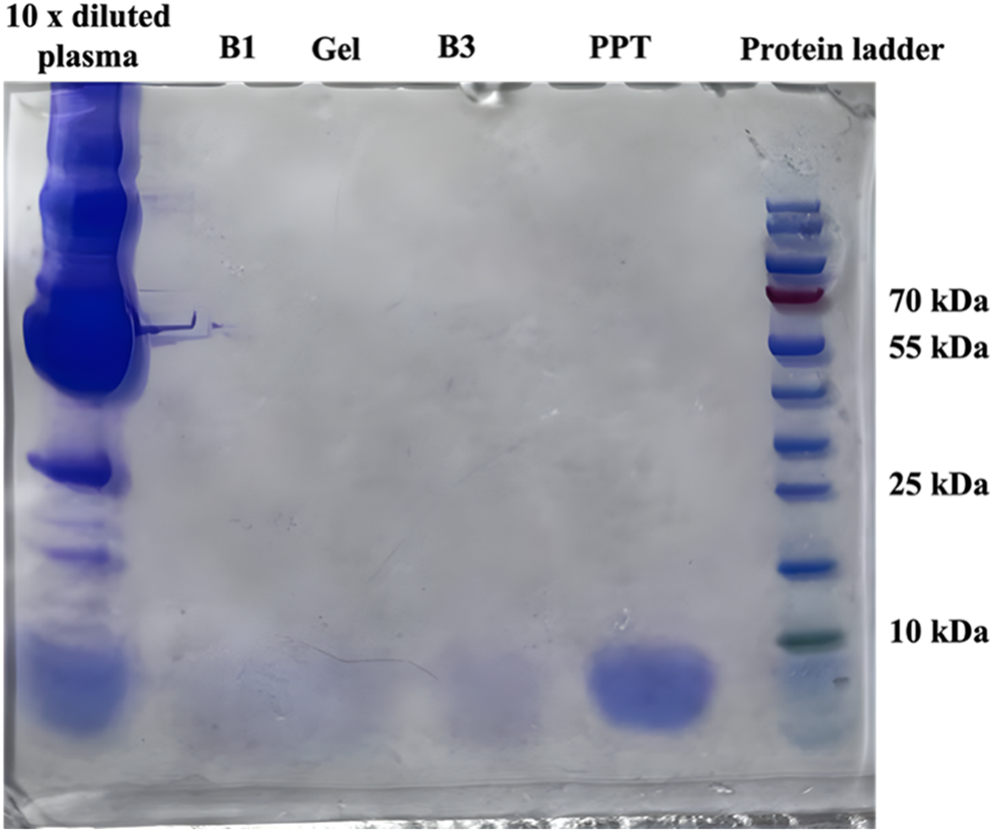
SDS PAGE electrophoresis of proteins; Lane 1 was a plasma sample before extraction diluted by a factor of 10. Lanes 2–5 were acceptor after extraction with the NPOE system, agarose system, B3 system, and traditional protein precipitation with acetonitrile, respectively. Lane 6 was a protein ladder.

Finally, the agarose system was investigated with respect to phospholipids. A plasma sample was diluted 1:10 v/v with 100 mM formic acid and was processed with the agarose system. The acceptor was analyzed for phospholipids by UHPLC-MS/MS as illustrated in Figure 4. The acceptor solution was free from detectable phospholipids (phosphatidylcholines) after extraction. The phospholipids were at their isoelectric point in this experiment and were not prone to electrokinetic migration. The same experiment was conducted in the NPOE and B3 systems, and the results were similar to those of the agarose system. Thus, all three systems were highly efficient with respect to the removal of phospholipids.

**Figure 4:**
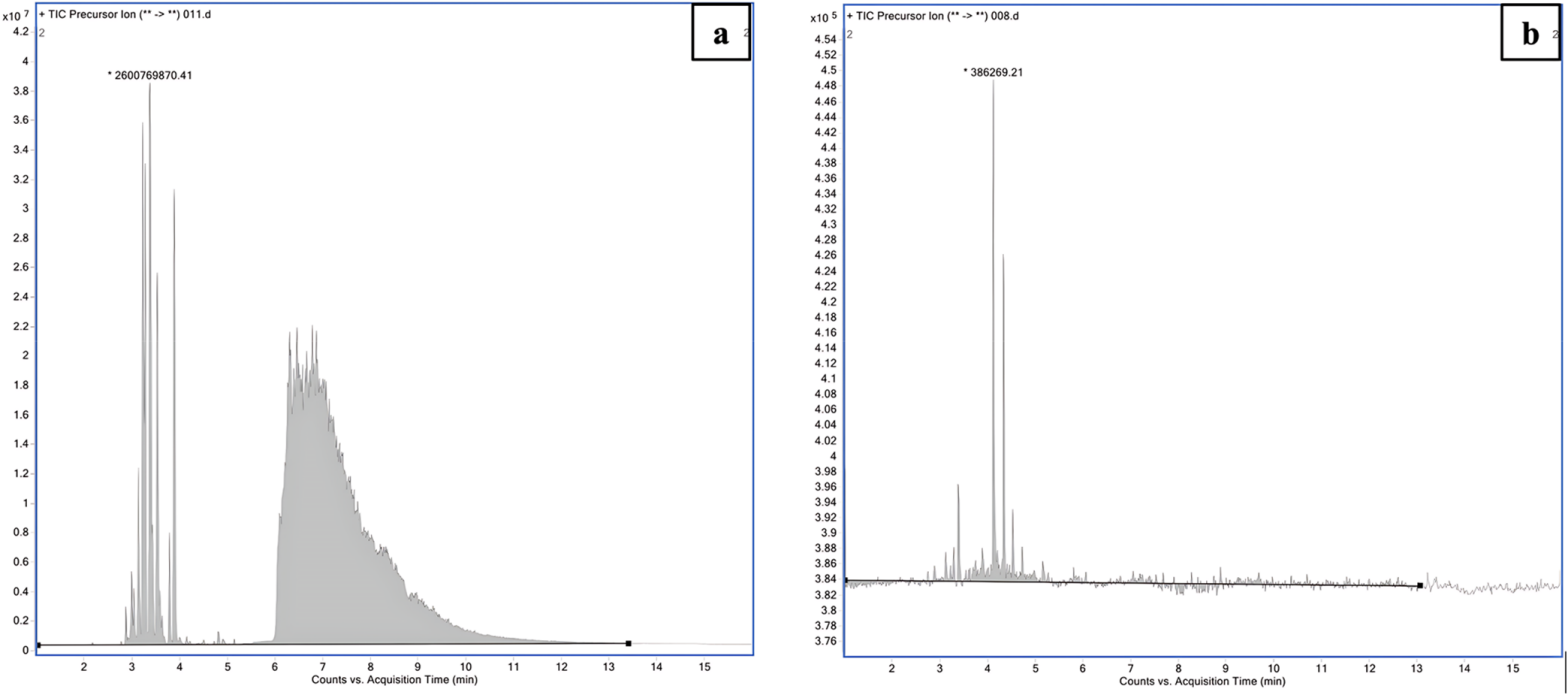
LC-MS/MS chromatogram of phospholipids (precursor scan mode, product ion *m*/*z* 184) in raw plasma sample (a) and acceptor after EME with agarose system (b).

### Selectivity and Capacity

From the data above (Figure 2a), extraction selectivity was clearly affected by the electrophoretic mobility of the analytes. With pH 4.0 in the sample, zwitterionic compounds such as sulfadiazine and bumetanide were close to their isoelectric point (net charge close to zero), and their electrokinetic migration into acceptor was more or less zero. This was also the case for very weak bases such as isoniazid. Selectivity was also affected by analyte interactions with agarose and hydrophilic PVDF. Generally, for compounds with log *P* > 3.0 (such as reserpine, promazine, chlorpromazine, chlorprothixene, and pimozide), recoveries decreased with log *P*, and we attributed this to hydrophobic interactions with agarose and PVDF in the aqueous environment. Compounds such as cimetidine, famotidine, and timolol with high hydrogen bond donor/acceptor count were extracted with low recoveries, and we attributed this to hydrogen bond interactions. Although extraction data were collected for a large number of substances, further generalizations were difficult due to multiple interactions involved.

In addition to the electrokinetic mobility and interactions with agarose and PVDF, electrochemical degradation may have affected some of the compounds. Dopamine and metaraminol are very similar structures in terms of log *P*, aromatic ring count, and the number of hydrogen bond donors and acceptors. However, both oxygens of dopamine are phenolic, and dopamine is prone to electrochemical degradation. Recoveries were 29 and 89% for dopamine and metaraminol, respectively, and this difference was attributed to electrochemical degradation.

The selectivity of the solvent-based systems B1 and B3 was different and was due to electro-assisted partition in a three-phase system (water–oil–water). Selectivity in the NPOE system was largely explained by log *P*, while that in the B3 system was more complex. The capacity for plasma samples was significantly higher with the solvent-based systems because the current was much lower and because most of the sample matrix was discriminated by the sample/liquid membrane interface.

## Conclusions

For the first time, gel electromembrane extraction was performed in a 96-well system, where the sample and acceptor were separated by an agarose-gel membrane (agarose system). The performance of the agarose system was compared to traditional electromembrane extraction systems with organic solvents as liquid membrane (solvent-based systems). The agarose system was efficient for basic pharmaceuticals in the polarity range −4.0 < log *P* < 3.0. Compounds with log *P* > 3.0 were discriminated due to hydrophobic interactions with the agarose-gel membrane and with the support membrane (hydrophilic PVDF). Inorganic ions passed the gel membrane, while proteins and phospholipids were discriminated. Therefore, the agarose system provided relatively clean extracts.

The agarose system was completely aqueous and provided substantial selectivity. The operation was challenged by high current and electroendosmosis, and this limited the extraction potential (voltage) and time. In addition, the capacity of plasma samples was limited. On the other hand, the agarose system has potential for very polar substances, and sophistication of the gel membrane may be used for tuning the selectivity. Such systems are aqueous, and this is an advantage in terms of predictability and sustainability.

EME was commercialized recently,^25^ and the number of scientific papers on this technique is increasing rapidly. The current paper investigated both solvent- and gel-based systems from a fundamental point of view, and the understanding from this is highly important for the further development and implementation of EME.

## Supplementary Material



## Notes

Model analytes, log *P* values, LC-MS parameters, extraction recoveries, and recoveries as a function of log *P* (PDF)
