# Supplementary material for: 96-Well Agarose-Gel Electromembrane Extraction
Source: Anal Chem. 2025 Dec 22;98(1):1090–7. doi: 10.1021/acs.analchem.5c06783 (PMC12809633; doi:10.1021/acs.analchem.5c06783)
Supplement: Supplementary file 1 [file ac5c06783_si_001.pdf]

Supporting Information

96-Well Agarose-Gel Electromembrane Extraction

Thidarat Samkumpim[1,2], Samira Dowlatshah[1,3], Waleed Alahmad[2], Pakorn Varanusupakul[2], Helena Hruskova[4], Frederik André Hansen[1], Stig Pedersen-Bjergaard[1,5]

[1]Department of Pharmacy, University of Oslo, 0316 Oslo, Norway

[2]Department of Chemistry, Chulalongkorn University, Patumwan Bangkok 10330, Thailand

[3]Extraction Technologies Norway, Verkstedveien 29, 1424 Ski, Norway

[4]Institute of Analytical Chemistry of the Czech Academy of Sciences, Brno 602 00, Czech Republic

[5]Department of Pharmacy, Faculty of Health and Medical Sciences, University of Copenhagen, Universitetsparken 2, 2100 Copenhagen, Denmark

Table of contents

Figure S1	Recovery versus log P for 90 basic model analytes with NPOE system
FigureS2	Recovery versus log P for 90 basic model analytes with B3 system
Table S1	Model analytes, producer, and LC-MS parameters
Table S2	Log P and extraction recoveries for all model analytes

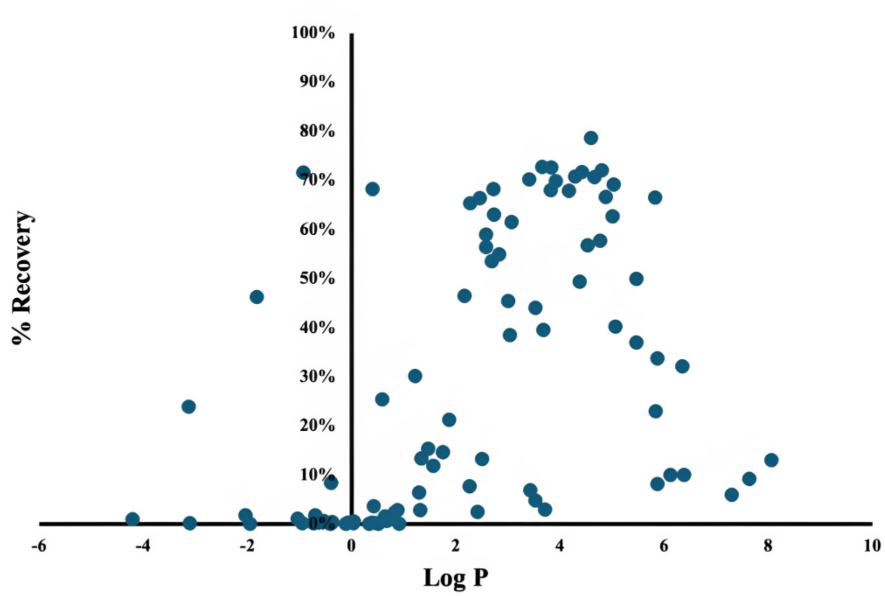

Figure S1. Recovery versus log P for 90 basic model analytes with NPOE system

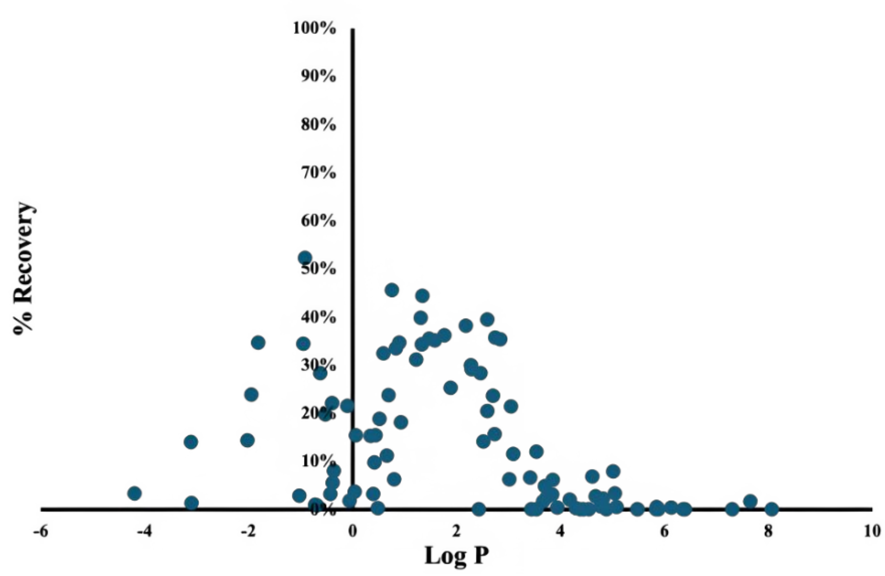

Figure S2. Recovery versus log P for 90 basic model analytes with B3 system

Table S1. Model analytes, producer, and LC-MS parameters.

Compound	Producer	Retention time (min)	Precursor Ion (m/z)	Product Ion (m/z)	Collision energy (V)
**2,6-di-tert-butyl-4-(dimethylaminomethyl)-phenol**	Sigma–Aldrich (St. Louis, MO, USA)	4.62	264.2	219.2 (quantifier)	17
			264.2	203.2	33
**6-MAM**	Sigma–Aldrich (St. Louis, MO, USA)	2.43	328.2	211.1	29
			328.2	165.1 (quantifier)	49
**Acetyl choline**	Sigma–Aldrich (St. Louis, MO, USA)	0.34	146	87 (quantifier)	13
			146	60.1	9
**Adenine**	Sigma–Aldrich (St. Louis, MO, USA)	0.35	136.1	119 (quantifier)	25
			136.1	65	45
**Alprenolol**	Sigma–Aldrich (St. Louis, MO, USA)	3.93	250.41	173.1	17
			250.41	116.1 (quantifier)	17
**Amantidine**	Sigma–Aldrich (St. Louis, MO, USA)	2.55	152.1	135.1 (quantifier)	17
			152.1	77	50
**Amiodarone**	Sigma–Aldrich (St. Louis, MO, USA)	6.39	646	276.1	45
			646	201.1 (quantifier)	37
**Amitriptyline**	Sigma–Aldrich (St. Louis, MO, USA)	4.67	278.2	105	25
			278.2	91 (quantifier)	29
**Antipyrin**	Sigma–Aldrich (St. Louis, MO, USA)	3.15	189.1	77 (quantifier)	49
			189.1	58.1	25
**Atenolol**	Sigma–Aldrich (St. Louis, MO, USA)	1.15	267.2	190.1	21
			267.2	145.1 (quantifier)	29
**Atropine**	Sigma–Aldrich (St. Louis, MO, USA)	2.78	290.41	124.1 (quantifier)	25
			290.41	93	33
**Benzamidine**	Sigma–Aldrich (St. Louis, MO, USA)	0.5	121.1	104 (quantifier)	21
			121.1	77	33
**Bumetanide**	Sigma–Aldrich (St. Louis, MO, USA)	6.04	365.1	240.1 (quantifier)	17
			365.1	184.1	25
**Butylhydrazine**	Sigma–Aldrich (St. Louis, MO, USA)	0.44	89.1	33.2 (quantifier)	9
			89.1	29.2	21
**Chlorpromazine**	Sigma–Aldrich (St. Louis, MO, USA)	4.86	319.1	246	25
			319.1	214.1 (quantifier)	49
**Chlorprothixene**	Sigma–Aldrich (St. Louis, MO, USA)	4.93	316.1	271 (quantifier)	21
			316.1	231	33

**Cimetidine**	Sigma–Aldrich (St. Louis, MO, USA)	1.05	253.1<br>253.1	159.1<br>95 (quantifier)	13<br>33
**Cinnarizine**	Sigma–Aldrich (St. Louis, MO, USA)	5.5	369.51<br>369.51	167.1 (quantifier)<br>152.1	21<br>50
**Clofazimine**	Merck (Darmstadt, Germany)	6.2	473.1<br>473.1	431 (quantifier)<br>395.1	41<br>50
**Clomipramine**	Sigma–Aldrich (St. Louis, MO, USA)	4.98	315.2<br>315.2	242.1<br>227 (quantifier)	29<br>45
**Clotrimazole**	Sigma–Aldrich (St. Louis, MO, USA)	4.97	277<br>277	241.1<br>165.1 (quantifier)	30<br>30
**Cocaine**	Norsk Medisinaldepot AS (NMD, Oslo, Norway)	3.33	304.2<br>304.2	182.1 (quantifier)<br>105	21<br>37
**Diphenylamine**	Sigma–Aldrich (St. Louis, MO, USA)	6.83	170.1<br>170.1	93<br>65.1	33<br>41
**Denatonium**	Sigma–Aldrich (St. Louis, MO, USA)	4.27	326<br>326	234.2 (quantifier)<br>112.1	17<br>29
**Diltiazem**	Sigma–Aldrich (St. Louis, MO, USA)	4.3	415.2<br>415.2	178 (quantifier)<br>150.1	25<br>49
**Dopamine**	Sigma–Aldrich (St. Louis, MO, USA)	0.41	154.1<br>154.1	137.1 (quantifier)<br>91	9<br>25
**Doxepin**	Sigma–Aldrich (St. Louis, MO, USA)	4.22	280.2<br>280.2	107 (quantifier)<br>91	25<br>49
**Droperidol**	Sigma–Aldrich (St. Louis, MO, USA)	3.88	380.2<br>380.2	194.1 (quantifier)<br>165.1	13<br>29
**Enalapril**	Sigma–Aldrich (St. Louis, MO, USA)	3.99	377.51<br>377.51	234.1 (quantifier)<br>117	17<br>41
**Ephedrine**	Sigma–Aldrich (St. Louis, MO, USA)	1.35	166.1<br>166.1	148.1 (quantifier)<br>91	9<br>37
**Epinephrine**	Sigma–Aldrich (St. Louis, MO, USA)	0.34	184.1<br>184.1	166.1 (quantifier)<br>77	5<br>49
**Famotidine**	Sigma–Aldrich (St. Louis, MO, USA)	0.98	338<br>338	259.1<br>189 (quantifier)	9<br>21
**Fluoxetine**	Sigma–Aldrich (St. Louis, MO, USA)	4.83	310.1	148.1 (quantifier)	5
**Halofantrine**		6.53	500.4	142.1 (quantifier)	30

	Sigma–Aldrich (St. Louis, MO, USA)		500.4	100.1	30
**Haloperidol**	Sigma–Aldrich (St. Louis, MO, USA)	4.33	376.1	165.1 (quantifier)	25
			376.1	123	50
**Hydralazine**	Sigma–Aldrich (St. Louis, MO, USA)	0.6	161.21	89 (quantifier)	25
**Hydroxyzine**	Sigma–Aldrich (St. Louis, MO, USA)	4.61	375.2	201.1 (quantifier)	21
			375.2	166.1	50
**Ipratropium**	Sigma–Aldrich (St. Louis, MO, USA)	2.93	332	166.2 (quantifier)	29
			332	124.1	37
**Isoniazid**	Sigma–Aldrich (St. Louis, MO, USA)	0.35	138.1	121 (quantifier)	13
			138.1	79	33
**Lidocaine**	Sigma–Aldrich (St. Louis, MO, USA)	2.68	235.2	86.1 (quantifier)	17
**Loperamide**	Sigma–Aldrich (St. Louis, MO, USA)	5.24	478.11	267.2 (quantifier)	25
			478.11	210.1	50
**Luminol**	Sigma–Aldrich (St. Louis, MO, USA)	2.33	178.1	105 (quantifier)	33
			178.1	78	45
**L-thyroxine**	Sigma–Aldrich (St. Louis, MO, USA)	5.24	777.7	731.6	25
			777.7	633.7	25
**Meclizine**	Sigma–Aldrich (St. Louis, MO, USA)	5.75	391.2	201.1 (quantifier)	17
			391.2	166.1	50
**Mepiquat**	Sigma–Aldrich (St. Louis, MO, USA)	0.35	114	98.1 (quantifier)	29
			114	58.1	29
**Metaraminol**	Sigma–Aldrich (St. Louis, MO, USA)	0.5	168.21	150.1 (quantifier)	5
**Metformin**	Sigma–Aldrich (St. Louis, MO, USA)	0.33	130.1	71.1	25
			130.1	60.1 (quantifier)	13
**Methadone**	Norsk Medisinaldepot AS (NMD, Oslo, Norway)	4.71	310.2	265.2 (quantifier)	13
			310.2	105	33
**Metoprolol**	Sigma–Aldrich (St. Louis, MO, USA)	3.13	268.41	116.1 (quantifier)	17
			268.41	74.1	21
**Mianserin**	Sigma–Aldrich (St. Louis, MO, USA)	4.1	265.2	208.1 (quantifier)	21
			265.2	91	50
**N-acetylputrescine**	Sigma–Aldrich (St. Louis, MO, USA)	0.33	131.1	114.1 (quantifier)	9
			131.1	72.1	13

**N-guanylurea**	Sigma–Aldrich (St. Louis, MO, USA)	0.32	103.1<br>103.1	60.1 (quantifier)<br>43.1	9<br>37
**Nicotinamide**	Sigma–Aldrich (St. Louis, MO, USA)	0.44	123.1<br>123.1	80 (quantifier)<br>53.1	21<br>33
**Normetanephrine**	Sigma–Aldrich (St. Louis, MO, USA)	0.40	184.1<br>184.1	166.1<br>134.1	5<br>17
**Nortriptyline**	Sigma–Aldrich (St. Louis, MO, USA)	4.6	264.41<br>264.41	105<br>91 (quantifier)	21<br>25
**Noscapine**	Sigma–Aldrich (St. Louis, MO, USA)	3.65	414.2<br>414.2	353.1<br>220.1 (quantifier)	25<br>21
**O-desmethylvenlafaxine**	Sigma–Aldrich (St. Louis, MO, USA)	2.87	264.2<br>264.2	246.2 (quantifier)<br>107	9<br>41
**Oxprenolol**	European Pharmacopoeia Reference Standard	3.52	266.2<br>266.2	225.1<br>116.1 (quantifier)	13<br>17
**Papaverine**	Sigma–Aldrich (St. Louis, MO, USA)	3.59	340.41<br>340.41	324.1 (quantifier)<br>202.1	33<br>29
**Perphenazine**	Sigma–Aldrich (St. Louis, MO, USA)	4.61	404.2<br>404.2	171.1 (quantifier)<br>143.1	25<br>29
**Pethidine**	Norsk Medisinaldepot AS (NMD, Oslo, Norway)	3.39	248.31<br>248.31	220.2 (quantifier)<br>174.1	21<br>21
**Pimozide**	Sigma–Aldrich (St. Louis, MO, USA)	5.18	462.2<br>462.2	328.2 (quantifier)<br>147.1	33<br>41
**Piperazine**	Sigma–Aldrich (St. Louis, MO, USA)	0.28	87.1<br>87.1	44.1 (quantifier)<br>27.2	17<br>45
**Practolol**	Sigma–Aldrich (St. Louis, MO, USA)	1.68	267.2<br>267.2	190.1 (quantifier)<br>148.1	17<br>25
**Procaine**	Sigma–Aldrich (St. Louis, MO, USA)	1.51	237.31	100.1 (quantifier)	13
**Prochlorperazine**	Sigma–Aldrich (St. Louis, MO, USA)	4.72	374.1<br>374.1	141.2 (quantifier)<br>113.1	21<br>33
**Promazine**	Sigma–Aldrich (St. Louis, MO, USA)	4.42	285.1<br>285.1	180.1<br>86.1 (quantifier)	49<br>17
**Promethazine**	Sigma–Aldrich (St. Louis, MO, USA)	4.34	285.41<br>285.41	198<br>86.1 (quantifier)	25<br>13
**Propranolol**		3.88	260.2	183.1	17

	Sigma–Aldrich (St. Louis, MO, USA)		260.2	116.1 (quantifier)	17
**Pyridoxine**	Sigma–Aldrich (St. Louis, MO, USA)	0.4	170.1<br>170.1	152.1 (quantifier)<br>134.1	9<br>21
**Pyrilamine**	Sigma–Aldrich (St. Louis, MO, USA)	3.24	286.2<br>286.2	241.1<br>121.1 (quantifier)	9<br>25
**Quinine**	Sigma–Aldrich (St. Louis, MO, USA)	2.72	163.1<br>163.1	189.1 (quantifier)<br>117	13<br>41
**Raloxifene**	Sigma–Aldrich (St. Louis, MO, USA)	4.22	473.6<br>473.6	269<br>112.1 (quantifier)	37<br>30
**Ranitidine**	Sigma–Aldrich (St. Louis, MO, USA)	1.23	315.1<br>315.1	176.1 (quantifier)<br>130.1	17<br>25
**Reserpine**	Sigma–Aldrich (St. Louis, MO, USA)	4.97	609.3<br>609.3	397.2<br>195.1 (quantifier)	29<br>41
**Salbutamol**	Sigma–Aldrich (St. Louis, MO, USA)	0.93	240.31<br>240.31	222.1<br>148.1 (quantifier)	5<br>17
**Serotonin**	Sigma–Aldrich (St. Louis, MO, USA)	0.6	177.1<br>177.1	160.1 (quantifier)<br>115	5<br>29
**Sotalol**	Sigma–Aldrich (St. Louis, MO, USA)	1.01	273.01<br>273.01	255.1 (quantifier)<br>133.1	9<br>29
**Sulfadiazine**	Sigma–Aldrich (St. Louis, MO, USA)	1.93	251.06<br>251.06	156<br>92 (quantifier)	15<br>30
**Sulfamethazine**	Sigma–Aldrich (St. Louis, MO, USA)	2.99	279.1<br>279.1	124.1<br>92 (quantifier)	25<br>33
**Sulfamethoxazole**	Sigma–Aldrich (St. Louis, MO, USA)	3.67	254.1<br>254.1	156<br>92 (quantifier)	13<br>29
**Tamoxifen**	Sigma–Aldrich (St. Louis, MO, USA)	5.99	371.5<br>371.5	129.1<br>72.27 (quantifier)	25<br>20
**Telmisartan**	Sigma–Aldrich (St. Louis, MO, USA)	4.82	258.1<br>258.1	305.1<br>211 (quantifier)	9<br>9
**Thiamine**	Sigma–Aldrich (St. Louis, MO, USA)	0.29	265<br>265	144.1<br>122.1 (quantifier)	9<br>13
**Thioridazine**	Sigma–Aldrich (St. Louis, MO, USA)	5.2	371.2<br>371.2	258.1<br>126.1 (quantifier)	29<br>25
**Timolol**	Sigma–Aldrich (St. Louis, MO, USA)	3.04	317.2<br>317.2	261.1 (quantifier)<br>244.1	13<br>21

**Triclabendazole**	Sigma–Aldrich (St. Louis, MO, USA)	6.89	358.9	343.9	29
			358.9	273.9 (quantifier)	41
**Triisopropanolamine**	Sigma–Aldrich (St. Louis, MO, USA)	0.38	192.2	174.2 (quantifier)	13
			192.2	98.1	21
**Trimipramine**	Sigma–Aldrich (St. Louis, MO, USA)	4.76	295.2	193.1	49
			295.2	100.1 (quantifier)	17
**Tyramine**	Sigma–Aldrich (St. Louis, MO, USA)	0.48	138.1	121.1 (quantifier)	5
			138.1	77	33
**Tyrosine methyl ester**	Sigma–Aldrich (St. Louis, MO, USA)	0.97	196.1	136.1	13
			196.1	91 (quantifier)	37
**Venlafaxine**	Sigma–Aldrich (St. Louis, MO, USA)	3.63	278.2	260.2 (quantifier)	9
			278.2	121.1	33
**Verapamil**	Sigma–Aldrich (St. Louis, MO, USA)	4.69	455.3	303.2	25
			455.3	165.1 (quantifier)	29

Table S2. Log P and extraction recoveries for all model analytes.

Sample	log P	Basic pKa	Acidic pKa	Agarose system	Agarose system	NPOE system	B3 system
				Recovery from dilute formic acid	Recovery from diluted plasma	Recovery from plasma	Recovery from plasma
Acetyl choline	-4.2	Permanent charge		81 %	125 %	0 %	5 %
Mepiquat	-3.12	Permanent charge		78 %	248 %	3 %	19 %
Thiamine	-3.1	Permanent charge, 5.54		29 %	30 %	0 %	2 %
N-Guanylurea	-2.03	9.97	11.48	24 %	30 %	1 %	20 %
Famotidine	-1.95	0.2, 7.98	10.5	9 %	98 %	0 %	29 %
Ipratropium	-1.82	Permanent charge		85 %	83 %	70 %	40 %
N-acetylputrescine	-1.03	9.9		95 %	58 %	1 %	4 %
Pyridoxine	-0.95	5.19	9.00	96 %	79 %	0 %	48 %
Metformin	-0.92	10.27, 12.33		56 %	163 %	37 %	63 %
Piperazine	-0.73	5.18, 9.56		35 %	56 %	0 %	1 %
Isoniazid	-0.69	2.29, 3.33		25 %	43 %	3 %	2 %
Triisopropanolamine	-0.63	8.63		112 %	131 %	33 %	37 %
Adenine	-0.53	4.73	10.29	14 %	33 %	8 %	29 %
Epinephrine	-0.43	8.91	9.69, 12.65	484 %	22 %	0 %	5 %
Sotalol	-0.4	9.64	8.75	91 %	102 %	0 %	31 %
Nicotinamide	-0.39	3.63		91 %	136 %	20 %	10 %
Normetanephrine	-0.37	9.00	9.98	97 %	17 %	1 %	11 %
Cimetidine	-0.11	6.53		13 %	87 %	0 %	27 %
Luminol	-0.06	1.62	8.21, 11.21	2 %	0 %	0 %	3 %
Dopamine	0.03	9.31	9.99, 13.03	29 %	59 %	3 %	8 %
Metaraminol	0.05	9.00	9.64	89 %	78 %	0 %	22 %
Salbutamol	0.34	9.40		84 %	80 %	0 %	23 %
Sulfadiazine	0.39	2.01	6.99	4 %	2 %	1 %	5 %
Denatonium	0.41	Permanent charge		75 %	85 %	105 %	9 %
Atenolol	0.43	9.27		96 %	107 %	0 %	23 %
Serotonin	0.48	10.02	9.32	44 %	35 %	0 %	1 %
Butylhydrazine	0.51	0.32, 8.49		50 %	115 %	0 %	27 %
Enalapril	0.59	5.21	3.88	54 %	23 %	54 %	38 %
Sulfamethazine	0.65	2.01	6.99	15 %	9 %	5 %	18 %
Tyramine	0.68	9.48	10.16	84 %	52 %	1 %	32 %
Hydralazine	0.75	1.69, 3.90		8 %	26 %	5 %	49 %
Sulfamethoxazol	0.79	1.97	5.86	2 %	0 %	2 %	8 %
Practolol	0.83	9.27		97 %	97 %	34 %	41 %
Benzamidine	0.89	11.53		26 %	93 %	6 %	38 %
Tyrosine methyl ester	0.92	7.29	9.51	52 %	56 %	0 %	27 %

Antipyrin	1.22	0.49		4 %	0 %	62 %	35 %
6-MAM	1.3	8.47	9.35, 11.72	43 %	91 %	13 %	49 %
Ephedrine	1.32	9.52		89 %	131 %	8 %	40 %
Timolol	1.34	9.36		18 %	69 %	25 %	51 %
Amantidine	1.47	10.46		86 %	138 %	21 %	39 %
Atropine	1.57	9.19		93 %	110 %	21 %	41 %
Metoprolol	1.76	9.27		85 %	95 %	26 %	41 %
Procaine	1.88	2.70, 8.96		77 %	172 %	1 %	35 %
Oxprenolol	2.17	9.27		20 %	86 %	75 %	41 %
O-desmethylvenlafaxine	2.27	8.90	9.80	114 %	109 %	15 %	35 %
Cocaine	2.28	8.95		82 %	103 %	99 %	31 %
Bumetanide	2.42	2.59	3.99, 9.62	0 %	1 %	56 %	0 %
Pethidine	2.46	8.14		79 %	120 %	97 %	30 %
Quinine	2.51	4.42, 8.55		21 %	132 %	13 %	13 %
Noscapine	2.58	6.16		35 %	96 %	102 %	37 %
Propranolol	2.58	9.27		72 %	96 %	91 %	19 %
Alprenolol	2.69	9.27		51 %	94 %	86 %	23 %
Diltiazem	2.73	8.18		54 %	72 %	108 %	15 %
Venlafaxine	2.74	9.04		78 %	94 %	92 %	39 %
Lidocaine	2.84	7.75		92 %	113 %	91 %	40 %
Droperidol	3.01	6.75	12.92	29 %	62 %	90 %	5 %
Pyrilamine	3.04	4.12, 8.76		35 %	122 %	70 %	24 %
Papaverine	3.08	6.03		39 %	77 %	99 %	10 %
Hydroxyzine	3.41	3.12, 7.97		74 %	84 %	101 %	6 %
Diphenylamine	3.43	0.78		3 %	1 %	3 %	0 %
Ranitidine	3.53	8.30		7 %	48 %	5 %	19 %
Reserpine	3.53	7.02		6 %	27 %	100 %	0 %
Haloperidol	3.66	8.14		28 %	72 %	105 %	2 %
Perphenazine	3.69	3.44, 8.61		6 %	15 %	102 %	4 %
L-thyroxine	3.72	9.43	0.69, 7.43	0 %	5 %	25 %	3 %
Mianserin	3.83	7.32		69 %	72 %	100 %	3 %
Doxepin	3.84	9.06		77 %	79 %	103 %	5 %
Promazine	3.93	9.20		4 %	21 %	91 %	0 %
Fluoxetine	4.17	9.40		52 %	54 %	100 %	2 %
Promethazine	4.29	9.05		52 %	39 %	100 %	0 %
Prochlorperazine	4.38	3.67, 8.68		5 %	6 %	54 %	0 %
Nortriptyline	4.43	10.47		60 %	73 %	56 %	0 %
Chlorpromazine	4.54	9.20		6 %	7 %	71 %	0 %
2,6-di-tert-butyl-4-(dimethylaminomethyl)phenol	4.6	8.80	10.75	80 %	91 %	106 %	6 %
Trimipramine	4.67	2.87, 9.42		44 %	47 %	102 %	2 %
Loperamide	4.77	9.50		27 %	24 %	95 %	1 %
Amitriptyline	4.81	9.06		59 %	58 %	101 %	2 %

Clomipramine	4.88	2.49, 9.2		36 %	29 %	80 %	0 %
Methadone	5.01	9.12		40 %	50 %	100 %	6 %
Verapamil	5.04	9.61		34 %	60 %	112 %	3 %
Chlorprothixene	5.07	9.06		13 %	11 %	91 %	1 %
Raloxifene	5.47	8.38	8.99, 9.58	7624 %	5 %	78 %	0 %
Thioridazine	5.47	9.43		0 %	2 %	53 %	0 %
Pimozide	5.83	8.88		0 %	1 %	56 %	0 %
Clotrimazole	5.84	6.26		28 %	2 %	58 %	1 %
Cinnarizine	5.88	2.81, 7.90		5 %	1 %	65 %	0 %
Triclabendazole	5.88	4.54	10.46	0 %	0 %	19 %	0 %
Telmisartan	6.13	4.57, 5.86	3.62	5 %	11 %	54 %	0 %
Tamoxifen	6.35	8.76		0 %	2 %	30 %	0 %
Meclizine	6.39	3.11, 8.03		1 %	0 %	19 %	0 %
Clofazimine	7.3	2.24, 5.89		0 %	0 %	2 %	0 %
Amiodarone	7.64	9.08		1 %	3 %	24 %	2 %
Halofantrine	8.06	10.2		1 %	2 %	3 %	0 %
